# Supplementary material for: Interactions between callose and cellulose revealed through the analysis of biopolymer mixtures
Source: Nat Commun. 2018 Oct 31;9:4538. doi: 10.1038/s41467-018-06820-y (PMC6208431; doi:10.1038/s41467-018-06820-y)
Supplement: Supplementary file 1 — Supplementary Information [file 41467_2018_6820_MOESM1_ESM.pdf]

Supplemental Information

Interactions between callose and cellulose revealed through the analysis of biopolymer mixtures

Abou-Saleh et al.

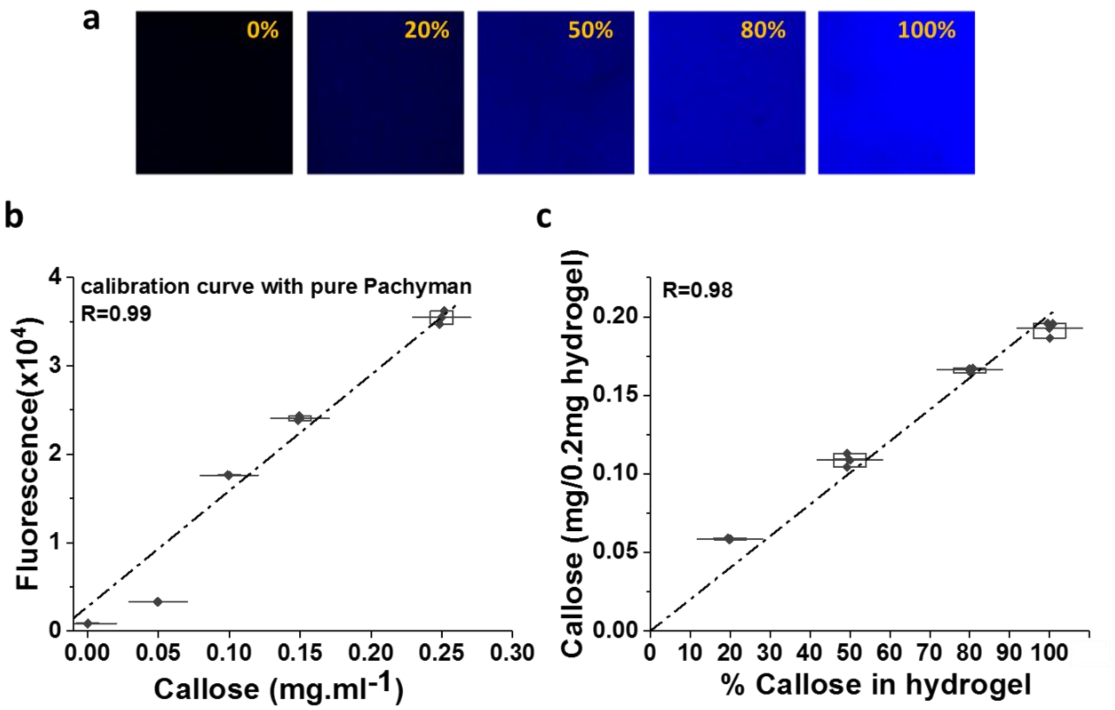

Supplementary Figure 1. Quantification of callose in composite hydrogels. **a**) Gels were stained with aniline blue and imaged using a confocal microscope Zeiss LSM700 (405nm excitation, 450nm emission). The panels show that fluorescence of the stained hydrogels increases with increasing percentage of callose (% indicated in the panels). **b**) Calibration curve showing relation between aniline blue fluorescence (arbitrary units recorded at 405nm excitation and 512nm emission) and weighted amounts of pachyman (callose analogue) dissolved in NaOH solution (mg ml^{-1}). **c**) Callose concentration (mg of pachyman per 0.2mg of hydrogels) was confirmed using fluorescence values and the calibration curve shown in **b**). Linear regression (R values) indicate good correlations between expected (x-axis) and experimental values (y-axis).). Boxes in **b** and **c** represent the first (25%) and third (75%) quartiles, the central line is the mean, and outliers at the 1% and 99% level are indicated by the whiskers, the data points are averages for 4 technical replicas.

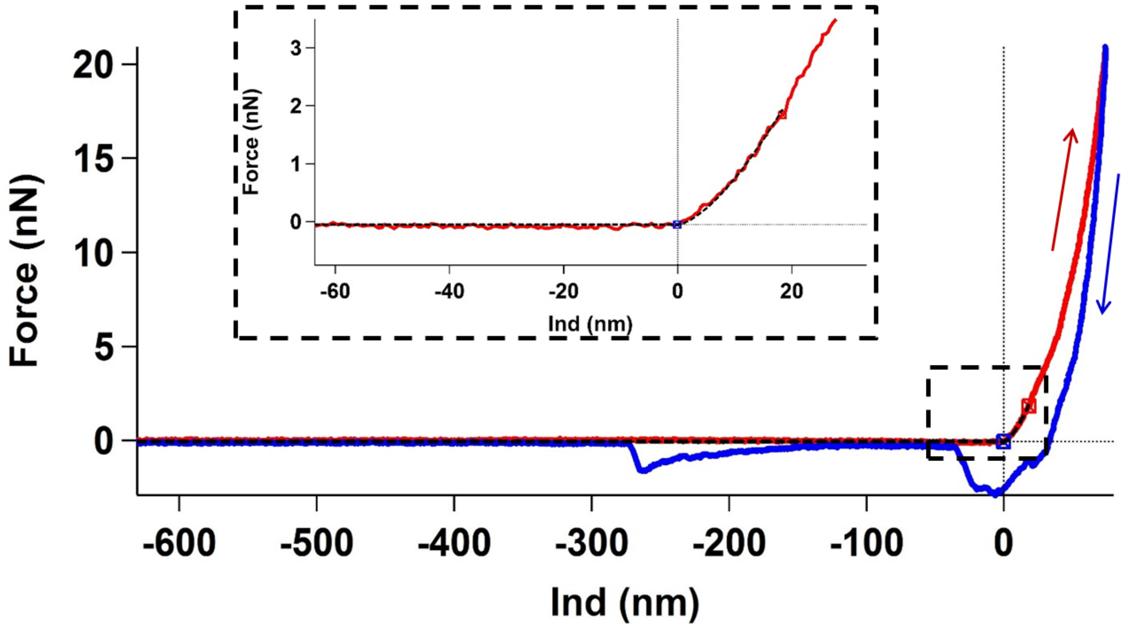

Supplementary Figure 2. The figure shows raw data for a typical force - deformation curve displaying the loading (red) and unloading (blue) curves. The discontinuous black line (over the loading curve around the contact point area, 0 nm) is shown in the inset and visualizes the Hertz model fitting for the 20% indentation only.

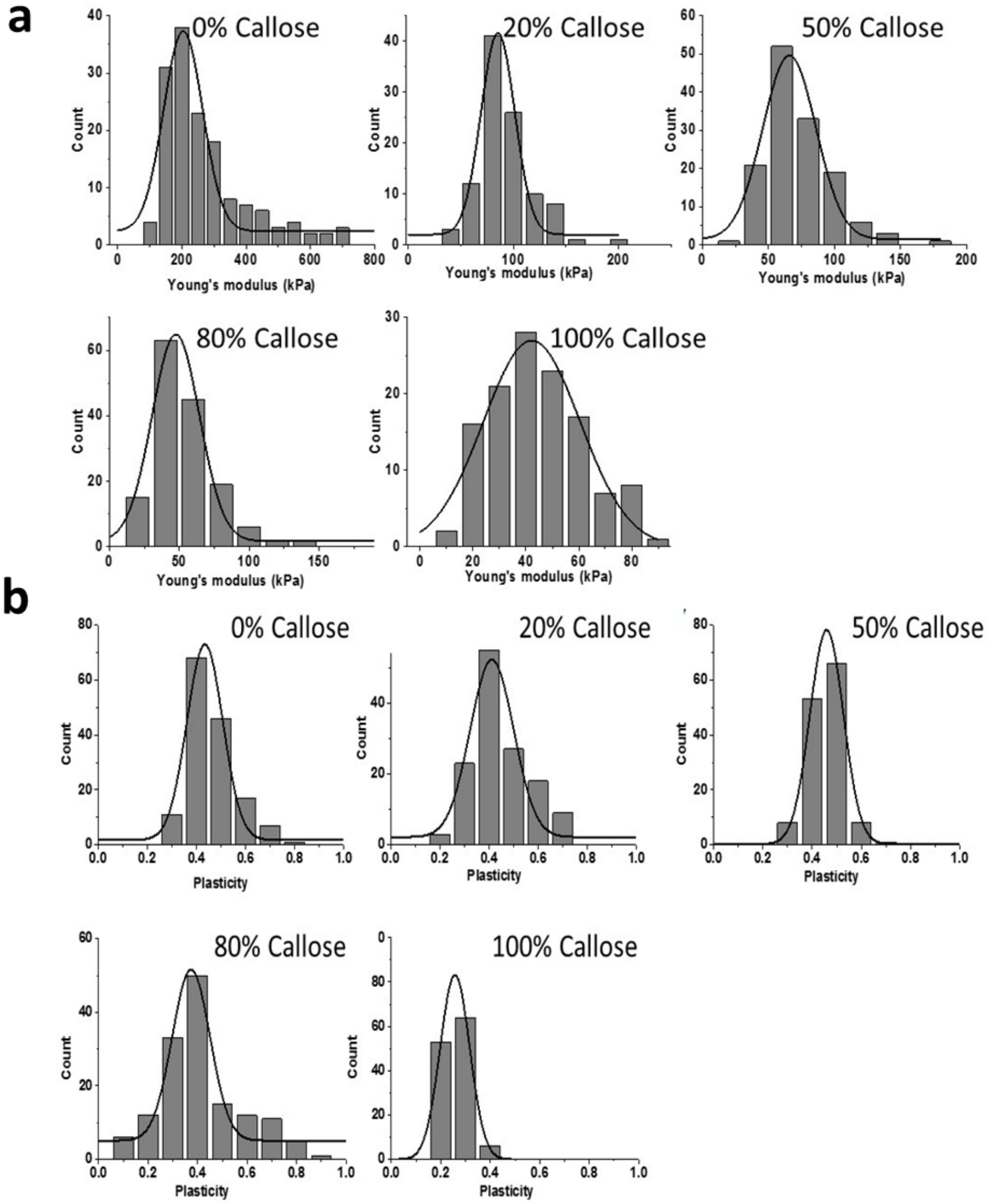

Supplementary Figure 3. Data analysis of AFM-nanoindentation results for the hydrogels. The figure shows one example (data distribution of only one volume map for each sample) from an average of 4 different areas that were used to calculate the average values for Young's modulus and the plasticity index displayed in Fig. 2. **a)** Histograms showing the Young's modulus repeats and distribution for hydrogels with different callose concentration. **b)** Histograms showing the plasticity index repeats and distribution for hydrogels with different callose concentration.

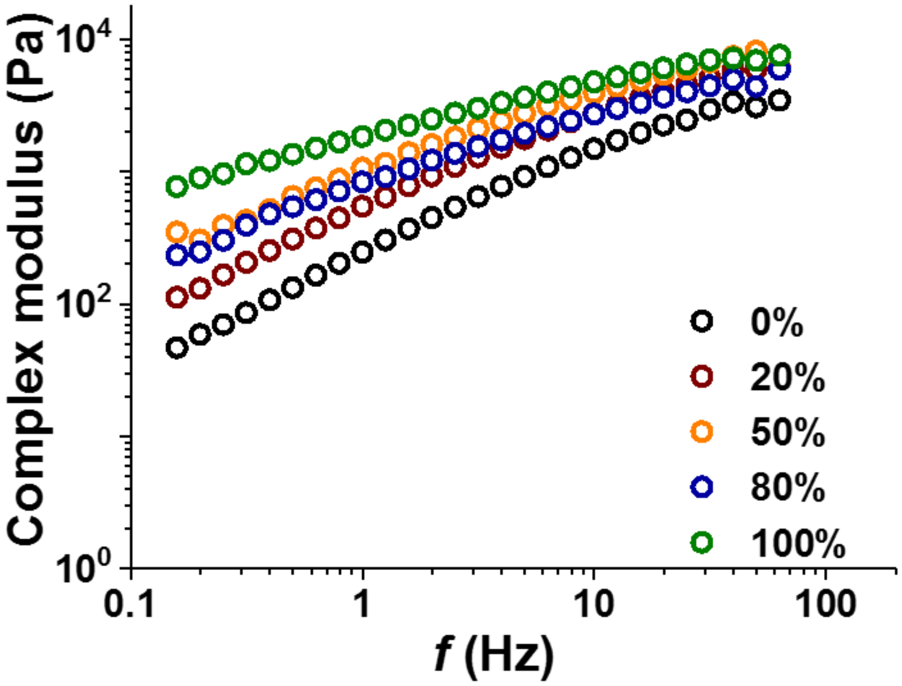

Supplementary Figure 4. The graph shows the complex modulus (*G**) as a function of applied oscillation frequency for the different compositions (percentage of callose). *G** increases with increasing callose concentration. Notice that values for 50% and 80% callose overlap, a pattern also observed in the zero shear rate viscosity values (Fig. 5).

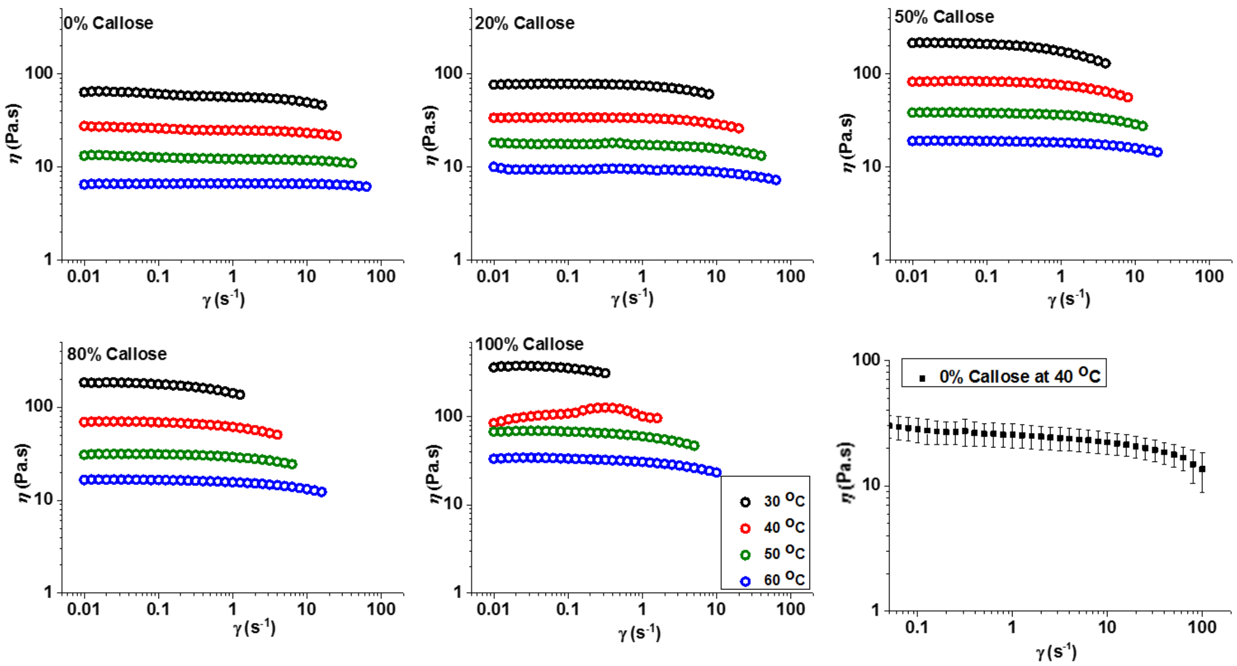

Supplementary Figure 5: Viscosity values as a function of frequency for the different callose concentration in the ionic liquid solutions. The figure shows that viscosity *η* increases with callose concentration and decreases within the temperature range 30-60 °C. Notice that viscosity is approximately constant at all shear rates γ suggesting Newtonian fluids. A graph for 0% callose at 40°C is shown to evaluate the dispersion in the curves. Error bars are SD from 3 replicas.

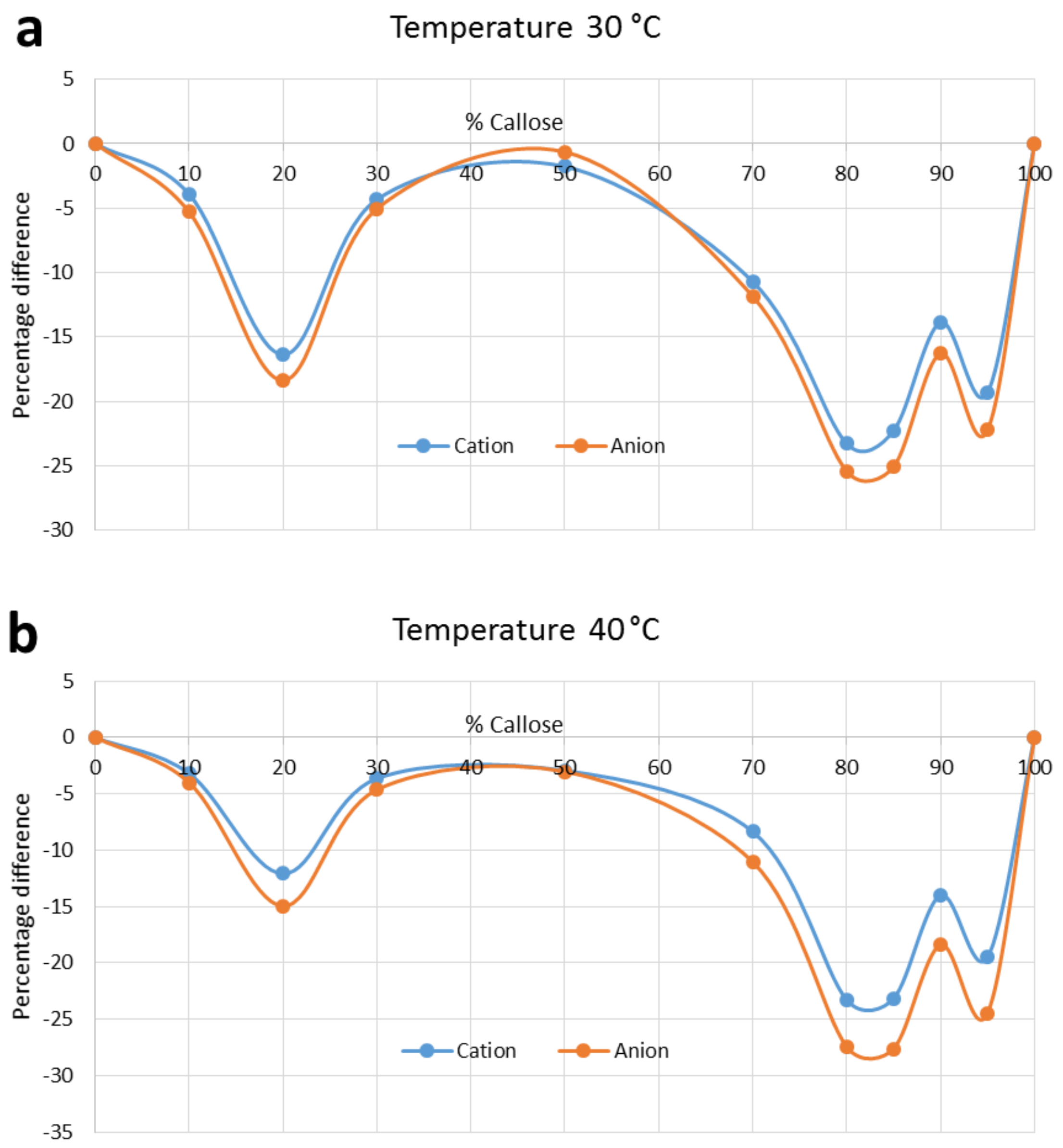

Supplementary Figure 6: The mixtures show non-ideal behaviours in the diffusion of the ions at different temperatures. The graphs represent the percentage difference relative to ideal values calculated assuming ideal mixing rule for the diffusion of the cation and the anion at different callose concentrations and at two different temperatures: **a**) shows at 30 °C **b**) shows at 40 °C. Notice that in both cases deviations to ideal mixing at 20% and about 80% callose concentrations.

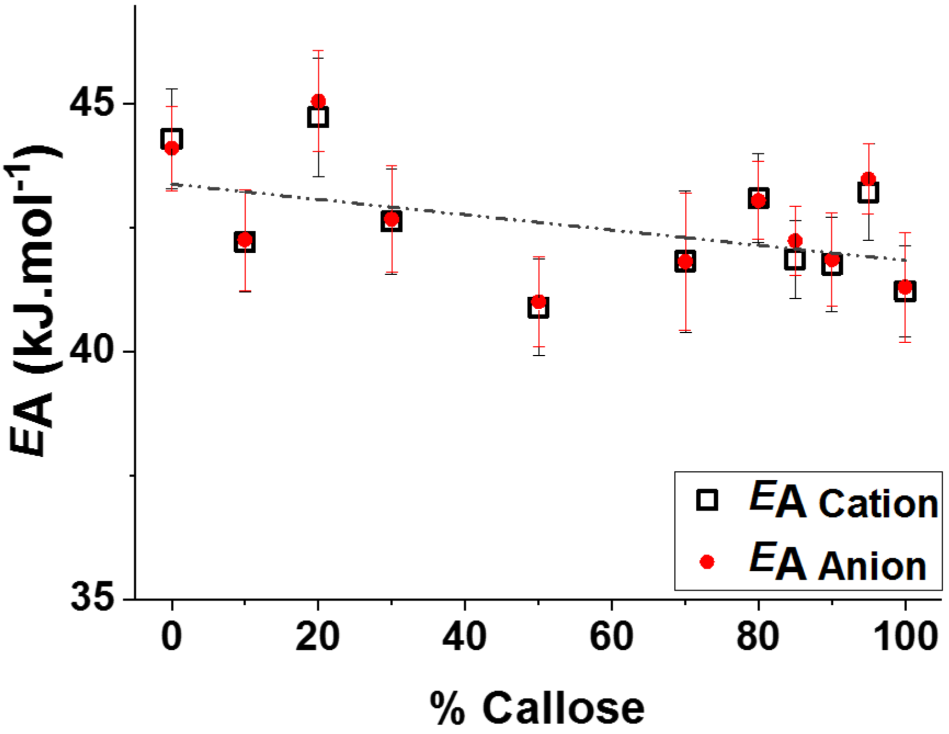

Supplementary Figure 7. Activation energy (E_A) as a function of callose concentration in the cellulose-callose mixtures at 30 °C. These were calculated using the Arrhenius equation from the anion and cation diffusion results obtained by NMR. The figure shows an overall small decrease with increasing the concentration of callose in relation to cellulose in the ionic solution. Error bars are standard deviations from three independent replicas.

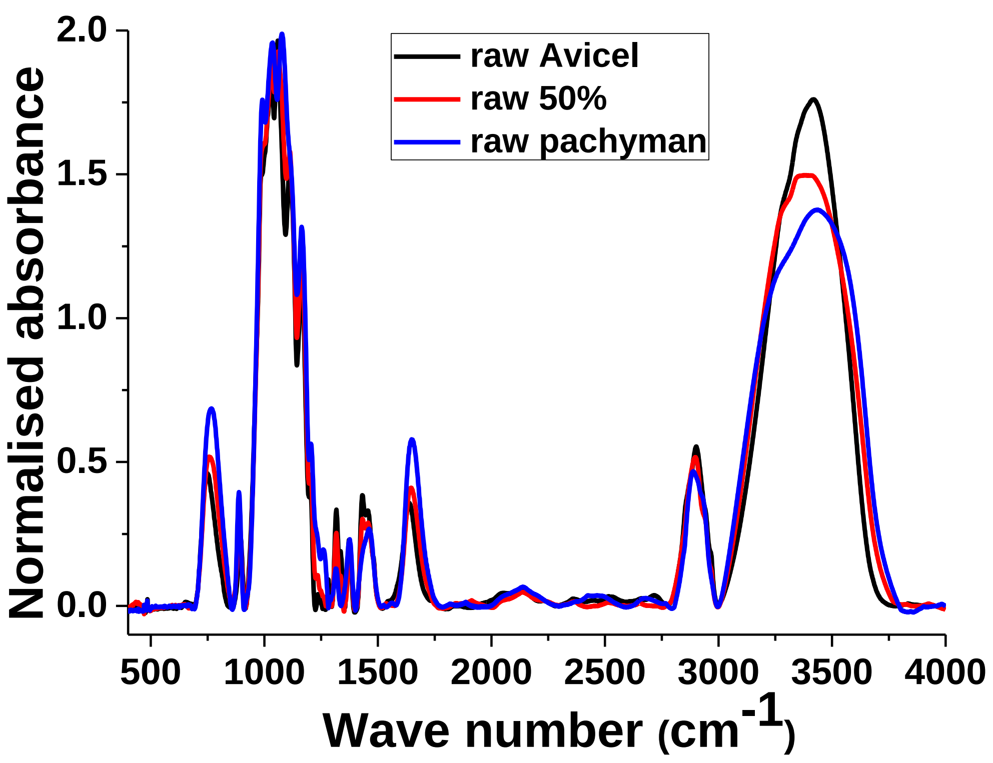

Supplementary Figure 8. Absorbance spectrum indicates similar profile for cellulose in commercial raw powder and plant tissues. The figure shows the FTIR spectra for dried Avicel and Pachyman powders and 50% Avicel – 50% Pachyman (raw 50%) powder mixtures in KBr discs. The position of distinctive peaks is mentioned in Supplementary Table 1.

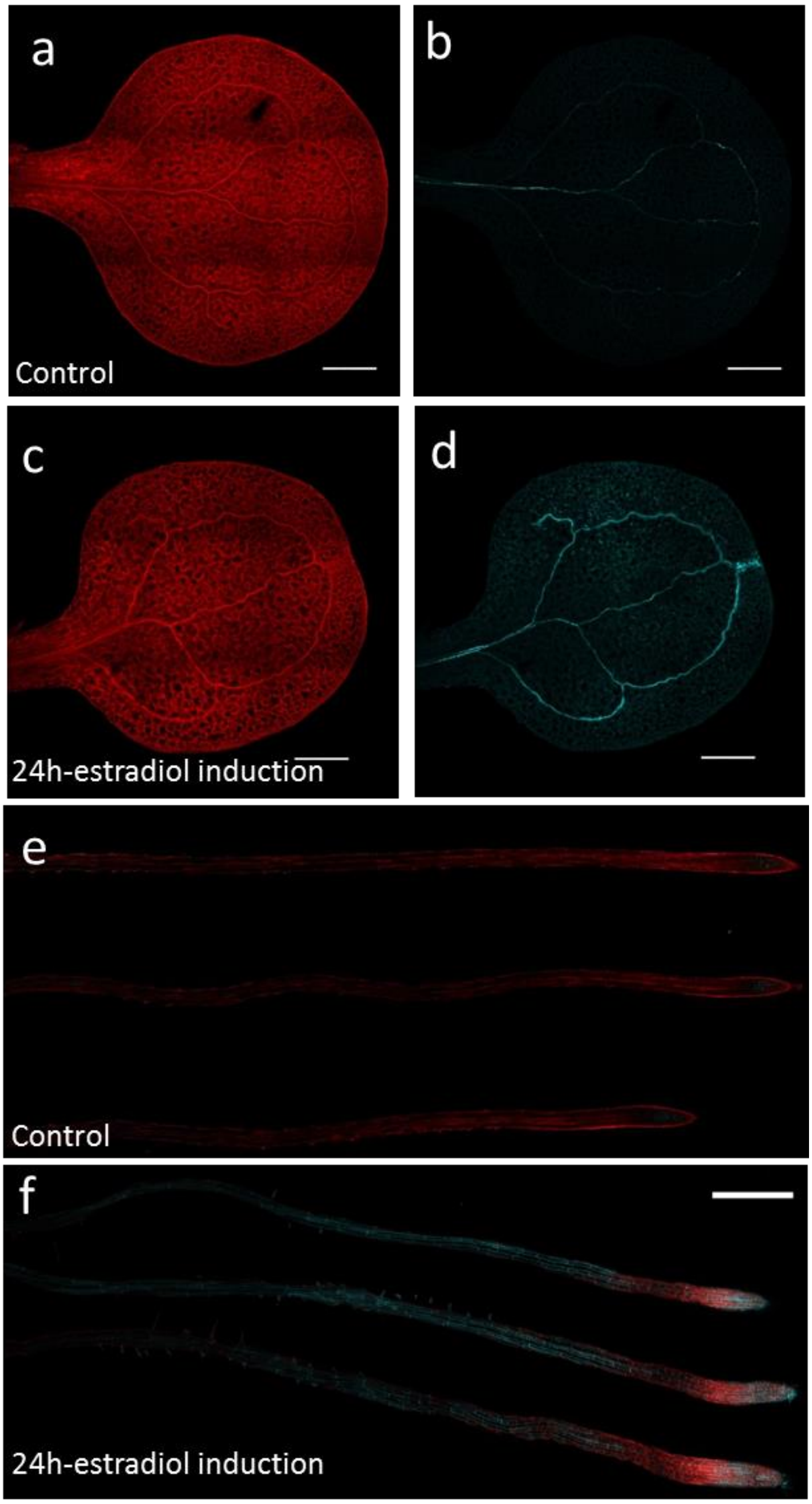

Supplementary Figure 9. Callose deposition patterns following 24 hours induction of the *pG1090::icals3m* line. DMSO-treated (control) and estradiol-treated *pG1090::icals3m* roots and leaves were co-stained with aniline blue and Direct Red 23 which fluorescently stain callose (cyan) and cellulose (red) respectively**. a-d**) shows confocal images of stained cotyledons in control and 24h after estradiol-induction in both the red (**a-c**) and cyan channels (**b-d**)**. e** and **f**) shows merged confocal images of stained roots after treatment with DMSO (control) and estradiol. Bars= 500 µm.

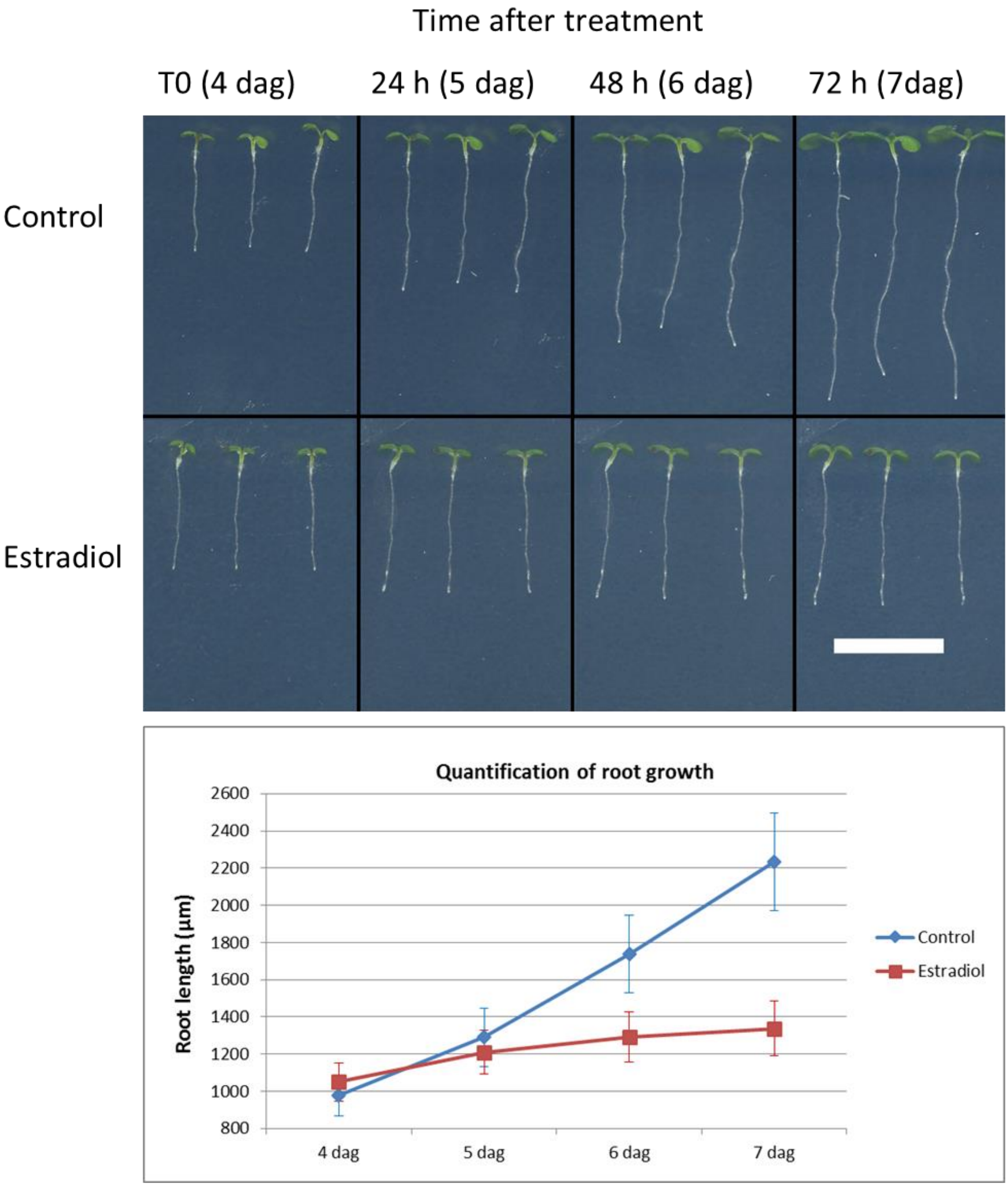

Supplementary Figure 10. Overall phenotypic characterization of the *pG1090::icals3m* line. **a**) Seedlings were pictured at T0 (no treatment, 4 days after germination, 4 dag), and at 24h (5 dag), 48h (6 dag) and 72h (7 dag) after treatment with DMSO (control, top panel) or estradiol (induction of callose, bottom panel). Bar= 1cm. **b**) The graph shows the root length measured following treatment with DMSO control or estradiol (n=10). Error bars are standard deviations. Differences were significant between control and estradiol treatment at 6 and 7 dag (p= 3.304×10^{-5} and 4×10^{-8} respectively) following a two tailed T-test.

Supplementary Table 1. Approximate position of key peaks for raw cellulose and wildtype plant cell wall samples obtained by FTIR. Related to Supplementary Figure 8, Figure 7 and Figure 9.

Wavenumber (cm^{-1})	Raw cellulose (Avicel)	Wildtype cell walls
*v*OH	3420	3350
*v*CH	2903	2930
*δ*CH2	1430	1445
*v*C–O–C	895	895

*v*OH indicates OH-stretching vibration, *v*CH: CH-stretching vibration, *δ*CH2: symmetric CH2 bending vibration, *v*C–O–C: corresponded to C–O–C stretching at glycosidic links.
